# High-resolution epitope mapping by AllerScan reveals relationships between IgE and IgG repertoires during peanut oral immunotherapy

**DOI:** 10.1016/j.xcrm.2021.100410

**Published:** 2021-10-19

**Authors:** Genghao Chen, Ellen L. Shrock, Mamie Z. Li, Jonathan M. Spergel, Kari C. Nadeau, Jacqueline A. Pongracic, Dale T. Umetsu, Rima Rachid, Andrew J. MacGinnitie, Wanda Phipatanakul, Lynda Schneider, Hans C. Oettgen, Stephen J. Elledge

**Affiliations:** 1 Department of Genetics, Harvard Medical School, Boston, MA 02115, USA; 2 Division of Genetics, Department of Medicine, Howard Hughes Medical Institute, Brigham and Women’s Hospital, Boston, MA 02115, USA; 3 Program in Biological and Biomedical Sciences, Harvard University, Cambridge, MA 02115, USA; 4 Division of Allergy and Immunology, Children’s Hospital of Philadelphia, Philadelphia, PA, USA; 5 Department of Pediatrics, Perelman School of Medicine of University of Pennsylvania, Philadelphia, PA, USA; 6 Department of Medicine, Sean N Parker Center for Allergy and Asthma Research, Stanford University, Palo Alto, CA, USA; 7 Division of Pediatric Allergy and Immunology, Feinberg School of Medicine, Northwestern University, Chicago, IL 60611, USA; 8 Ann & Robert H. Lurie Children’s Hospital of Chicago, Chicago, IL 60611, USA; 9 Division of Immunology, Department of Pediatrics, Boston Children’s Hospital, Boston, MA 02115, USA; 10 Department of Pediatrics, Harvard Medical School, Boston, MA 02115, USA

**Keywords:** peanut allergy, antibody, antibody repertoire, phage display, oral immunotherapy, food allergy, IgE, epitope mapping, high-throughput sequencing, allergen immunotherapy

## Abstract

Peanut allergy can result in life-threatening reactions and is a major public health concern. Oral immunotherapy (OIT) induces desensitization to food allergens through administration of increasing amounts of allergen. To dissect peanut-specific immunoglobulin E (IgE) and IgG responses in subjects undergoing OIT, we have developed AllerScan, a method that leverages phage-display and next-generation sequencing to identify the epitope targets of peanut-specific antibodies. We observe a striking diversification and boosting of the peanut-specific IgG repertoire after OIT and a reduction in pre-existing IgE levels against individual epitopes. High-resolution epitope mapping reveals shared recognition of public epitopes in Ara h 1, 2, 3, and 7. In individual subjects, OIT-induced IgG specificities overlap extensively with IgE and exhibit strikingly similar antibody footprints, suggesting related clonal lineages or convergent evolution of peanut-specific IgE and IgG B cells. Individual differences in epitope recognition identified via AllerScan could inform safer and more effective personalized immunotherapy.

## Highlights

• Phage immunoprecipitation sequencing simultaneously maps IgE and IgG peanut epitopes
• Ara h 1, 2, 3, and 7 epitopes are highly shared among allergic individuals
• High-resolution epitope mapping is achieved by saturation mutagenesis
• Oral immunotherapy induces IgG specificities overlapping with pre-existing IgE

## Graphical abstract

**Figure:**
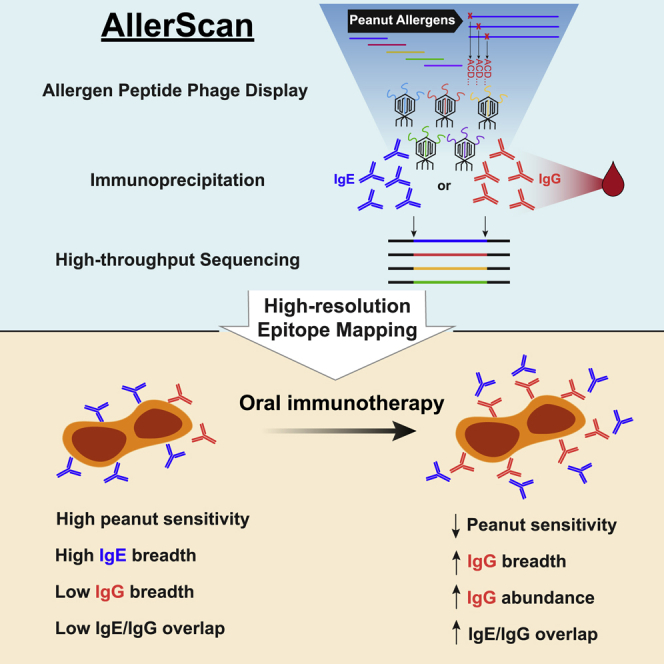

## Teaser

Chen et al. use a phage-display-based method to simultaneously map IgE and IgG epitopes in peanut-allergic patients undergoing oral immunotherapy (OIT). They identify shared epitopes and potentially therapeutically relevant individual differences in epitope recognition. OIT boosts and diversifies IgG responses that overlap with IgE, often with similar critical residues.

## Introduction

Peanut allergy is emerging as a growing health challenge in children, currently affecting 2.2% of the pediatric and 1.8% of the adult populations in the United States.^1^^,^^2^ Individuals with peanut allergy harbor immunoglobulin E (IgE) antibodies directed against peanut component proteins. Bound to the high-affinity IgE receptor FcεRI on the cell surface, IgE antibodies trigger the activation of tissue-resident mast cells and circulating basophils upon allergen encounter. This leads to the release of vasoactive mediators, including histamine, that cause allergic reactions. The most severe clinical manifestation of IgE-mediated allergy is systemic anaphylaxis, a life-threatening response that compromises multiple organ systems, including the respiratory and cardiovascular systems. The fact that not all subjects who are sensitized (i.e., who produce IgE antibodies) to food allergens exhibit allergic reactions suggests that there are factors that counteract IgE-mediated responses. Antibodies of the IgG class, which increase during the natural resolution of food allergies, may play an important role in this regard^3^, ^4^, ^5^ and have been shown to suppress IgE-induced allergic reactions by (1) competing with IgE for binding allergen epitopes,^6^ (2) accelerating allergen clearance by forming immune complexes, and (3) preventing mast cell activation by binding to the inhibitory IgG receptor, FcγRIIb.^6^, ^7^, ^8^, ^9^

Oral immunotherapy (OIT), the administration of gradually increasing daily doses of a food allergen over several months, has emerged in recent years as a promising approach for inducing desensitization to peanut in allergic children. However, the mechanisms underlying successful induction of the peanut unresponsive state are incompletely understood. Investigations of immune responses during OIT have revealed decreases in peanut-specific IgE concentration and increases in peanut-specific IgG concentration in the serum, suggesting two possible mechanisms that might contribute to desensitization.^10^^,^^11^ We and others have shown that these OIT-induced IgG antibodies suppress IgE-mediated basophil activation triggered by peanut allergens,^8^^,^^12^ indicating an important suppressive function of the IgG response induced by OIT. Although peanut OIT has now been approved for clinical use for treatment of peanut allergy, the therapy has significant limitations. Patients undergoing OIT commonly experience gastrointestinal symptoms and allergic reactions.^13^ Further, the food unresponsive state induced by OIT is transient and children who do not continue regular ingestion of the allergenic food following completion of the therapy exhibit a high rate of reversion to their initial allergic state.^14^ A recent meta-analysis of OIT studies revealed increased risk of allergic reactions and anaphylaxis and little improvement to quality of life in participants despite successful desensitization in most.^13^ A more detailed understanding of the modulation of the peanut-specific IgE and IgG antibody repertoires during OIT might inform safer and more effective regimens in the future.

Our group has previously developed phage-display-based platforms for autoantigen discovery^15^ (PhIP-seq) and profiling of antiviral humoral immunity (VirScan).^16^ To address questions about antibody repertoire changes during OIT, we developed AllerScan, an adaptation of PhIP-seq for detecting antibody responses to allergens—in this case, peanut. AllerScan enables comprehensive detection of IgE and IgG linear epitopes within allergenic peanut proteins. By profiling antibody responses to peanut peptides in OIT participants, we uncover characteristic changes in IgE and IgG repertoires that could explain the induction of tolerance. High-resolution antibody profiling through saturation mutagenesis shows conserved immunodominant “public” epitopes among allergic individuals and suggests clonal relationships between pre-existing IgE and OIT-induced IgG specificities.

## Results

### Development of the peanut AllerScan library

**Figure 1:**
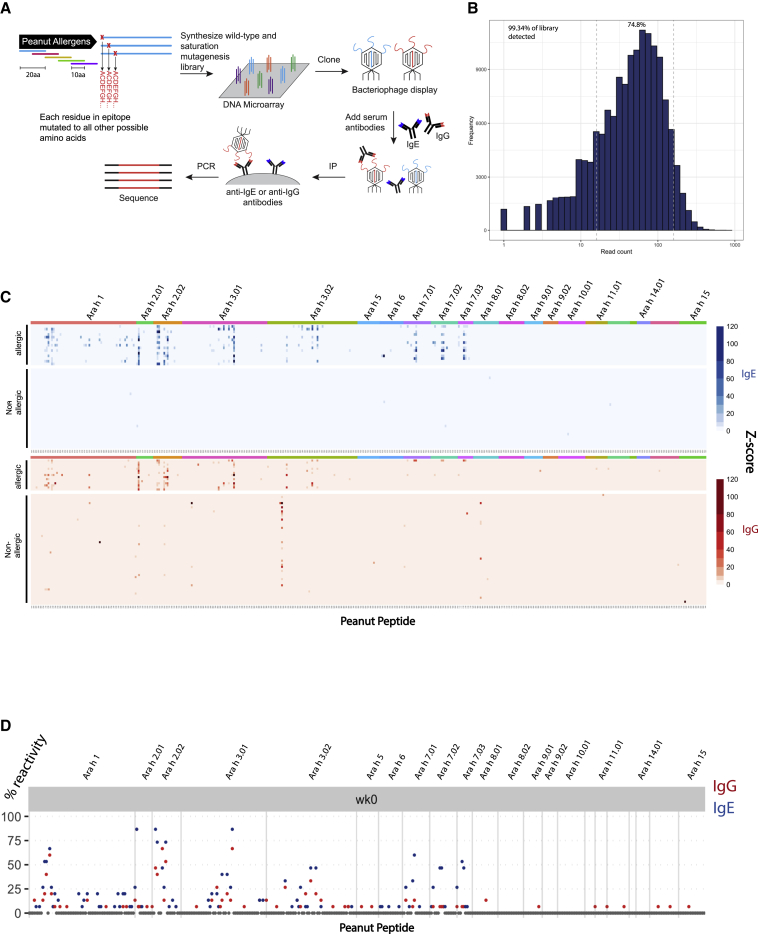
AllerScan reveals epitope targets of anti-peanut IgE and IgG antibodies in allergic patients and controls (A) Construction of peanut AllerScan library and overview of the AllerScan workflow. The peanut AllerScan library consists of 20-mer peptides tiling across the sequences of all peanut allergen proteins as well as saturating mutant versions of the peptides. DNA oligonucleotides encoding these peptides were synthesized and cloned into a phage-display library. To perform an AllerScan reaction, serum is mixed with the AllerScan phage library, IgE or IgG antibodies are immunoprecipitated, and bound phage is sequenced to identify the displayed peptides. (B) Distribution of the peanut AllerScan library. At approximately 60-fold sequencing coverage, 99.34% of library members were detected and 74.8% were within one-log abundance. (C) Heatmaps depict the IgE (blue) and IgG (red) antibody response to peanut peptides in allergic patients (n = 15) and healthy controls (n = 30 for IgE; n = 54 for IgG). Each row represents a sample from a unique individual. Each column represents a wild-type peptide from the peanut allergen protein labeled at top. The color intensity indicates the *Z* score representing the level of enrichment of the peptide. *Z* scores are averages of 2 replicates. (D) Seroprevalence of antibodies to peanut epitopes among allergic individuals. y axis shows percentage of allergic patients exhibiting IgE (blue) and IgG (red) reactivity to peptides in the peanut AllerScan library. Baseline allergic sera were collected at week 0 of the PRROTECT study.

To map epitopes targeted by the antibody response to peanut in allergic subjects, we constructed a phage-display library of 397 20-mer peptides tiling every 10 amino acids across the sequences of all 12 peanut allergens listed in the World Health Organization and International Union of Immunological Societies (WHO/IUIS) Allergen Nomenclature Sub-committee database at the time of library design (Table S1). Genetic and splice variants of the allergen proteins were also included (Figure 1A). In order to map epitopes with single-amino-acid resolution, we additionally designed 20-mer peptides that achieved saturation mutagenesis of the 397 wild-type peanut peptides, such that each residue of the wild-type peptides was substituted with every other possible amino acid at that position. To assess the quality of the constructed peanut AllerScan library, we sequenced the library at approximately 60-fold coverage. 99.34% of designed peptides were recovered, and 74.8% were within one-log abundance, demonstrating that our library is complete and evenly distributed (Figure 1B). To perform an AllerScan reaction, we mixed this peanut saturation mutagenesis phage-display library with serum, immunoprecipitated IgE or IgG antibodies, and sequenced bound phage to identify the peptides recognized by antibodies. In this study, we used peanut AllerScan to analyze the IgE and IgG repertoires in participants of a peanut OIT clinical trial, PRROTECT^17^ (details of the study described in STAR Methods section) and healthy controls.

### AllerScan of allergic sera identifies shared recognition of public epitopes and individualized binding patterns

To test whether we could detect peanut-specific antibody responses using AllerScan, we screened 15 serum samples collected from peanut-allergic (PA) individuals at week 0 of PRROTECT (before OIT) and 54 serum samples from non-allergic (NA) control donors (Figure 1C). We profiled IgE and IgG responses in all the allergic subjects and in 30 of the 54 NA donors. As a result of limited sample volume, we profiled only IgG responses in the remaining 24 NA donors. As expected, none of the 30 NA donors exhibited IgE responses to any of the peanut peptides in the library. In contrast, we detected clear IgE responses to peanut peptides in all of the PA patients (Figure 1C). The pattern of peptide binding varied among individuals, but overall, we detected strong IgE responses targeting the major peanut allergens Ara h 1, Ara h 2, Ara h 3, and Ara h 7; some IgE responses to Ara h 6; and little to no IgE recognition of other Ara h proteins. We detected most previously reported linear IgE epitopes in Ara h 1, Ara h 2, Ara h 3, and Ara h 7, including ELQGDRRCQSQLERA and RRCQSQLERANLRPC in Ara h 2 and EDEYEYDEEDRRRGR in Ara h 3 reported by Santos et al.^18^ (Table S2). This validated the robustness of AllerScan in reproducing the findings of orthogonal serological assays.^18^, ^19^, ^20^ In addition, we found some peptides to be highly “public,” i.e., bound by IgE from a large fraction of allergic patients (Figures 1C and 1D). Specifically, based on a *Z* score cutoff of 3.5, 20 peptides were recognized by >30% of patients; 10 peptides were recognized by >50% of patients; and 5 peptides were recognized by >70% of patients. The most public epitopes, Ara h 2.02 amino acid (aa)21–40, Ara h 3.01 aa301–320 and Ara h 2.01 aa61–80, were recognized by IgE in 13/15 patients. We also detected IgE responses to 59 additional “private” epitopes, recognized by <30% of allergic patients (Table S2).

We observed IgG responses to peanut peptides in allergic patients as well as in some NA donors, but the diversity of peptides recognized by NA individuals was much lower (Figure S1A). On average, NA donors exhibited IgG responses to 2 peptides per individual while allergic subjects from the PRROTECT study exhibited IgG responses to 7 peptides per individual. Interestingly, we found that one public epitope, Ara h 3.02 aa81–100, was recognized by 35% (19/54) of NA donors but 0% (0/15) of the allergic subjects. Furthermore, we found only a relatively small overlap between the NA and PA IgG epitopes. Overall, only 7 out of 34 IgG epitopes shared by at least 2 NA donors were also shared by any PA sera (Figure S1B). This suggests that different IgG epitopes are associated with allergic and NA phenotypes. In allergic subjects, IgG epitopes were found to be a subset of IgE epitopes and were recognized less frequently than IgE epitopes. For example, the most public IgE epitope, Ara h 2.02 aa21–40, was recognized by IgE in 13 patients but by IgG in only 7 patients (Table S3). In addition, 11 patients exhibited IgE reactivity to peptides from Ara h 7, but only 3 patients exhibited IgG reactivity.

**Figure 2:**
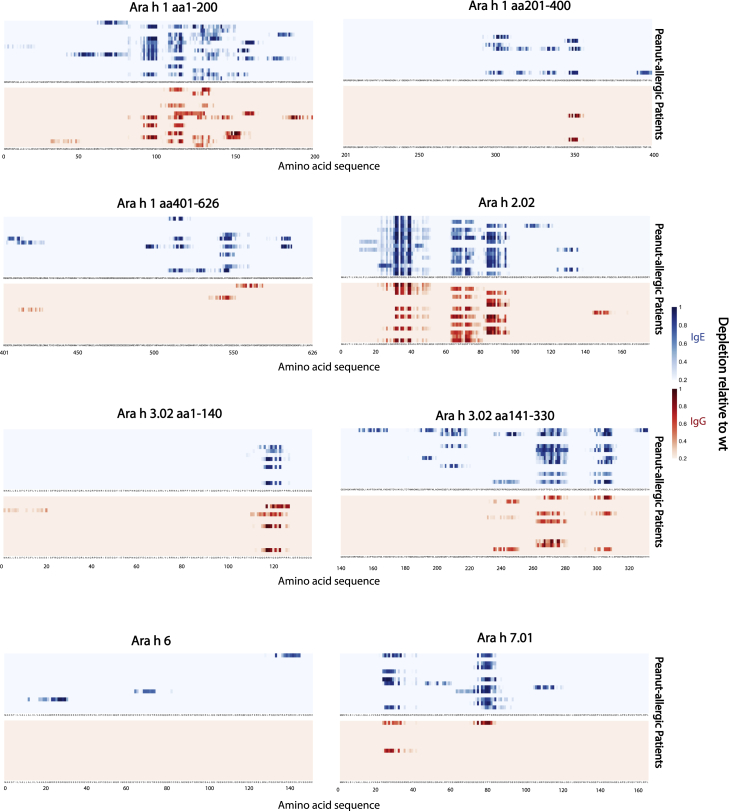
Mapping of IgE and IgG epitopes in major peanut allergens using saturation mutants Each column of the heatmap corresponds to an amino acid position; each row represents an allergic patient. The color intensity indicates the substitution effect at each amino acid position within the peanut allergens, blue representing IgE epitopes and red representing IgG epitopes (details in STAR Methods). Deeper shades represent greater disruption of antibody binding. For simplicity of presentation, results of only one of the technical replicates are shown.

To map the epitopes more precisely within the peanut peptides, we analyzed binding to saturation mutagenesis peptides to calculate a substitution score, or the average depletion of all mutants at each given amino acid position compared to the wild-type peptide. This analysis enabled us to identify the critical residues in the epitopes, as substitutions of these residues generally disrupt antibody binding. Consistent with our previous analysis of the wild-type peptides, the substitution scores indicated the presence of public epitopes in Ara h 1, 2, 3, and 7. For most public epitopes, various allergic patients exhibited similar patterns of critical residues (Figure 2). IgG exhibited similar critical residue recognition as IgE.

### Many individuals recognize public epitopes with similar antibody footprints

We next examined the antibody binding to the saturation mutant peptides in greater detail to determine whether the epitopes recognized by different patients on the same peptide were related. For each of the saturation mutant versions of a given peanut peptide, we calculated the relative depletion of the mutant compared to the wild-type peptide. We then generated a heatmap to illustrate the effect of each amino acid substitution on antibody binding. These heatmaps represented the “high-resolution antibody footprints” of an epitope.

**Figure 3:**
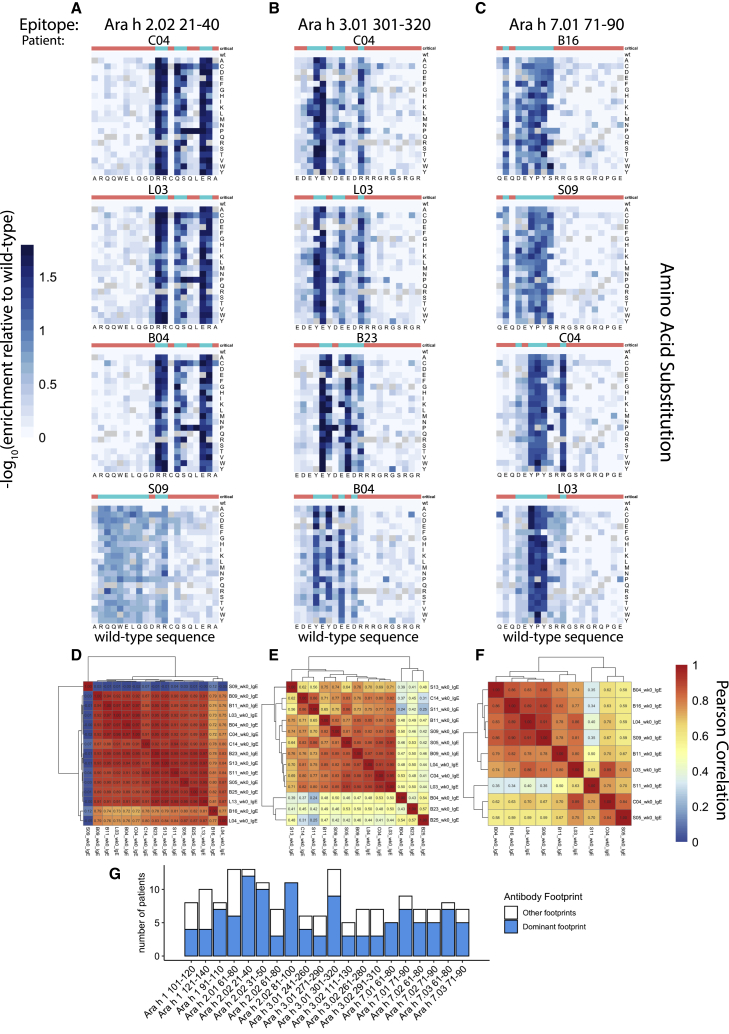
High-resolution profiling of antibody footprints reveals conserved public peanut epitopes (A–C) Representative examples of high-resolution antibody footprints from allergic patients (week 0 samples) for public peanut epitopes Ara h 2.02 aa21–40 (A), Ara h 3.01 aa301–320 (B), and Ara h 7.01 aa71–90 (C). Heatmaps plot the −log10 transformed relative enrichment compared to the adjusted wild-type value, which represents the substitution effects on antibody binding. x axis, amino acid sequence of the wild-type peanut epitope; y axis, amino acid substitutions. Critical residues for antibody binding are indicated at the top of each heatmap in blue; non-critical residues are indicated in red. (D–F) Pairwise Pearson correlations between high-resolution footprints of all allergic patients with antibody responses to the three peptides from (A)–(C), respectively. (G) Number of patients who share the dominant IgE antibody footprint for the public epitopes indicated by the x axis. Blue, patients who share the dominant footprint; white, patients with non-dominant footprints.

We selected three representative IgE public epitope peptides for further analysis: Ara h 2.02 aa21–40; Ara h 3.01 aa301–320; and Ara h 7.01 aa71–90, which were recognized by 13, 13, and 9 patients before OIT, respectively (Figures 3A–3C). For each of these peptides, we generated a clustered heatmap to illustrate the pairwise correlation coefficients between the high-resolution footprints of all patients who recognize the epitope (Figures 3D–3F). If we observed a correlation coefficient of >0.75 between 2 patients, we judged that these patients shared the same antibody footprint. A footprint was considered “dominant” if it was shared by the highest number of patients, regardless of whether the number reached a majority. To provide a reference, the correlation coefficients between antibody footprints from technical replicates of the same sera are often between 0.80 and 0.99. We found that the dominant RR-QS–ER motif in Ara h 2.02 aa21–40 was critical for antibody binding in 12 of 13 individuals with antibody responses to this peptide. Similarly, the YE-DE–R motif within Ara h 3.01 aa301–320 was critical in 9 of 13 individuals, and E-DEYPYS within Ara h 7.01 aa71–90 was critical for 6 of 9 individuals.

Despite the fact that, frequently, a large proportion of patients who recognized a public epitope peptide shared the same critical residues, some individual footprints deviated from the dominant pattern. For example, patient S09 recognized the N-terminal RQQWELQG-RR stretch in Ara h 2.02 aa21–40, while the dominant footprint recognized by all other 12 patients resided in the C-terminal RR-QS–ER stretch. For peptide Ara h 3.01 aa301–320, the differences between dominant and subdominant footprints were more subtle: the Y4 (tyrosine at position 4) was critical in the dominant footprint shared by 9 patients, while all substitutions except proline at this position were tolerated by patient B23. In contrast, Y6 was critical for B23 but dispensable for the dominant footprint. Uniquely for patient B04, both Y4 and Y6 were critical for antibody binding. As a final example, R11 mutations in Ara h 7.01 aa71–90 had little effect on IgE binding by patient B16 and 6 others that shared the dominant footprint, but this position was critical for patient C04. Sequence logo plots generated for the same epitopes also revealed these differences in antibody footprints among individuals (Figure S4A).^21^

To summarize, we found that, for 16/20 peptides that were recognized by IgE in >30% of the allergic subjects at week 0, a single dominant footprint was shared by ≥50% of individuals (Figure 3G). The presence of these highly similar footprints among individuals suggests that different individuals generate antibodies to peanut epitopes that are very similar at their epitope-binding interfaces.

### Peanut OIT results in a diversification and boosting of the peanut-specific IgG repertoire

**Figure 4:**
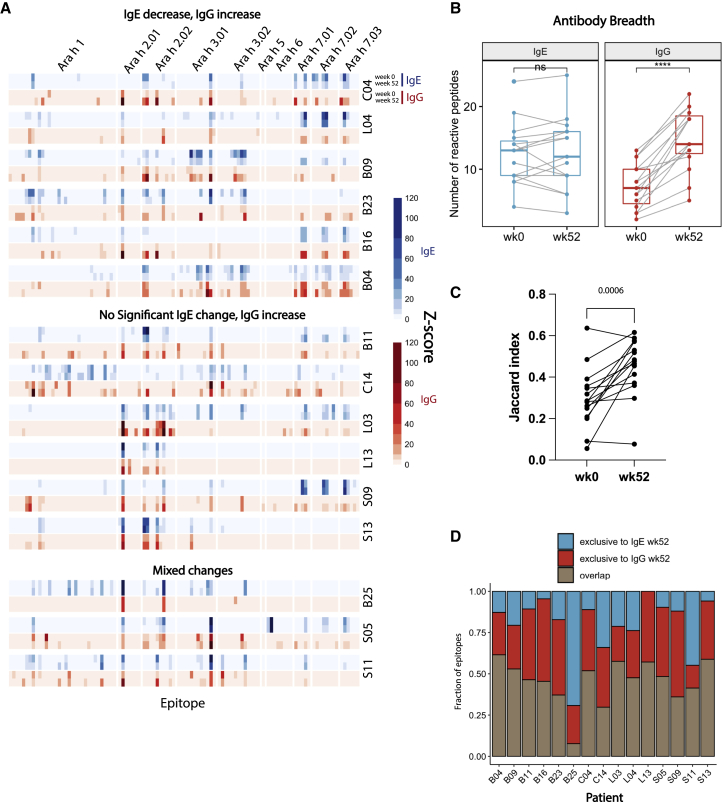
Peanut oral immunotherapy diversifies peanut-specific IgG repertoire (A) Heatmaps depict IgE (blue) or IgG (red) binding signal to individual wild-type peptides before (week 0, upper) and after (week 52, lower) OIT. Patients were stratified into 3 categories, depending on the type of IgE and IgG binding change. Top: patients exhibiting overall IgE binding decrease and IgG increase are shown; middle: patients with unchanged IgE but increased IgG are shown; bottom: patients with mixed changes in IgE and IgG binding are shown. (B) Boxplots depict the number of IgE (blue) and IgG (red) peptides recognized by each allergic patient before and after OIT (n = 15 biological replicates). Only peptides from 1 variant of each Ara h protein were included in the calculation. Wilcoxon signed rank test was used to determine statistical significance. (C) Jaccard index representing IgE and IgG repertoire overlap at week 0 and week 52 of OIT. p value indicated on top was calculated using Wilcoxon matched-pair signed rank test. (D) Overlap between IgE and IgG epitopes at week 52 of OIT. y axis shows each of the following categories in fraction of total epitope: blue, epitopes exclusively recognized by the IgE; red, epitopes exclusively recognized by IgG; brown, epitopes recognized by both the IgE and IgG.

Although peanut OIT has shown some promise in the clinic in recent years, the specific changes to the antibody repertoire induced by OIT remain incompletely understood. A 3 to 4 log induction of allergen-specific IgG levels occurs during OIT, but important questions remain about whether this reflects a more diversified IgG repertoire or simply an expansion of pre-existing IgG specificities. Therefore, we applied AllerScan to a cohort of allergic subjects sampled pre- and post-OIT to dissect IgE and IgG repertoire changes with high resolution (Figure 4A).

We first evaluated the overall breadth of peanut-specific IgE and IgG repertoires pre- and post-OIT. To this end, we counted the number of wild-type peptides recognized by IgG and, separately, IgE in each patient sample at week 0 (pre-OIT) and at week 52 (post-OIT). To avoid counting homologous peptides encoded by different protein variants multiple times, we limited this analysis to peptides from only 1 variant of each Ara h protein. We found that IgE epitope diversity did not change significantly after OIT: patients exhibited IgE recognition of 14 peanut peptides on average both before and after OIT (Figure 4B). In contrast, IgG epitope diversity increased significantly after OIT: an average of 7 peanut peptides were recognized by patient IgG before OIT but 15 peptides after OIT (Figure 4B). Indeed, all 15 patients exhibited an expansion of the peanut-specific IgG repertoire as a result of OIT (Figure 4B). This expansion led to an increase in the overlap between IgE and IgG epitopes (Figure 4C) and the addition of novel exclusively IgG epitopes post-OIT (Figure 4D). IgG binding to the same epitopes as IgE could block IgE-mediated activation of effector cells via steric hindrance,^6^^,^^12^ whereas novel, exclusive IgG epitopes could provide protection through the inhibitory FcγRIIb receptor on effector cells.^6^, ^7^, ^8^, ^9^

**Figure 5:**
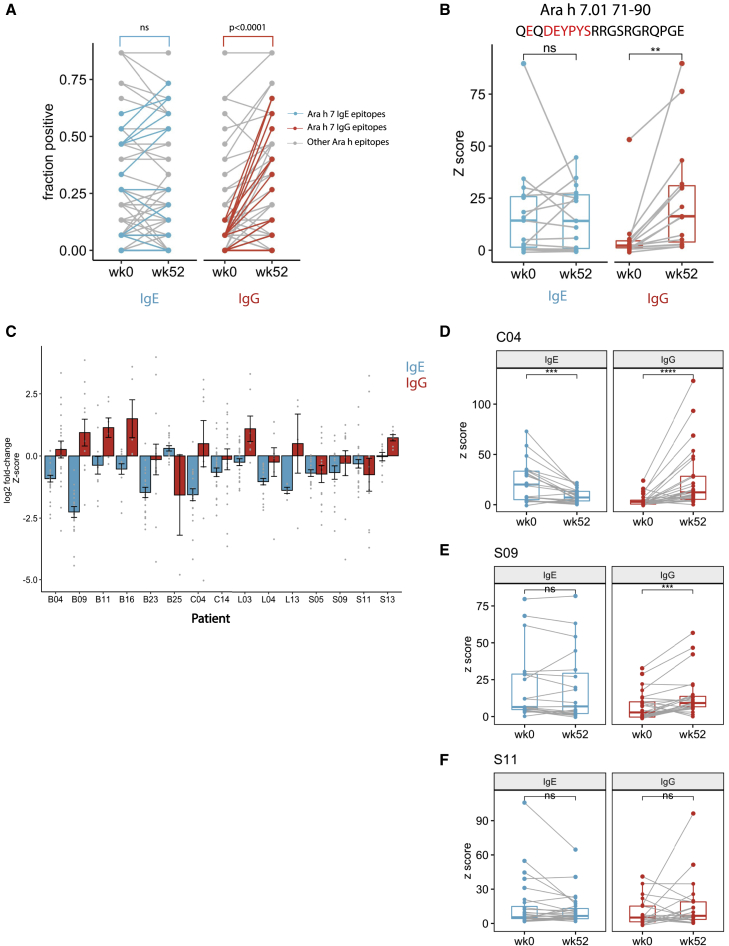
Peanut oral immunotherapy increases peptide-specific IgG levels while reducing abundance of pre-existing IgE specificities (A) Change in seroprevalence of IgE (left) and IgG (right) against individual epitopes after OIT. Ara h 7 epitopes are highlighted in blue (IgE) and red (IgG). p values were calculated by Mann-Whitney test. (B) Antibody binding *Z* score for a representative Ara h 7 epitope before (week 0) and after OIT (week 52). (Left) IgE *Z* scores are shown; (right) IgG *Z* scores are shown. Critical residues in the dominant footprint are highlighted in red. Each point represents one patient. Statistical significance was determined by paired t test. (C) Change in IgE (blue) and IgG (red) binding *Z* score to pre-existing epitopes after OIT. Each bar represents the mean log2 fold change of *Z* scores for all pre-existing epitope peptides (*Z* score > 3.5 at week 0) at week 52 versus at week 0 for the OIT subject indicated by the x axis. Error bars denote SEM. (D–F) Peptide binding *Z* scores changes of representative patients exhibiting both IgE decrease and IgG increase (D), IgE unchanged but IgG increase (E), and mixed changes in IgE and IgG (F). Only peptides with either week 0 or week 52 reactivity were examined. Each dot represents one peptide. Statistical significance was determined by Wilcoxon signed rank test. ∗p < 0.05; ∗∗p < 0.01; ∗∗∗p < 0.001; ns, not significant.

In addition, we noted a striking induction of IgG to multiple peptides from Ara h 7 after OIT (Figures 5A and S1C). Although before OIT, we detected IgG responses to Ara h 7 in only 2 patients, after OIT, 12 patients had detectable IgG binding to at least one Ara h 7 peptide. For example, IgG binding to Ara h 7.01 aa71–90 was only detectable in 2/15 pre-OIT sera but 10/15 post-OIT sera, with *Z* score increases of up to 25-fold (Figure 5B). These results suggest that Ara-h-7-specific IgG could be a feature of successful desensitization by OIT.

### Peanut OIT increases peanut-specific IgG abundance while decreasing IgE levels against pre-existing epitopes

Using the peptide *Z* score as a measure of peptide-specific antibody abundance in the serum, we observed a slight decrease in peanut-specific IgE and a striking increase in peanut-specific IgG abundance in post-OIT sera (Figures S6B and S6C). Because epitope recognition varied among OIT patients, we evaluated changes in peptide-specific IgE and IgG levels for each patient individually. When the combined set of epitopes from pre- and post-OIT sera for a given individual was considered, 6/15 patients exhibited overall decrease in anti-peanut IgE abundance and 12/15 patients showed increase in anti-peanut IgG (Figures 4A, 5D–5F, and S5). When only the pre-existing epitopes (recognized by pre-OIT sera) were evaluated for each patient individually, we found that 12/15 patients exhibited significant reduction in IgE binding (Figure 5C). This suggests that the OIT-induced decrease in total peanut-specific IgE reported by us and others^8^^,^^10^ is driven by the decreased abundance of pre-existing IgE specificities but relatively unchanged diversity (Figure 4B). On the other hand, the increase in total peanut-specific IgG after OIT is likely driven by a combination of newly emerged IgG specificities (Figure 4B) as well as an increase in pre-existing IgG (Figure 5C), which suggests that OIT might be conducive to IgG+ B cell plasma cell differentiation rather than IgE switching.

### IgE and IgG from the same individual exhibit highly similar antibody footprints

**Figure 6:**
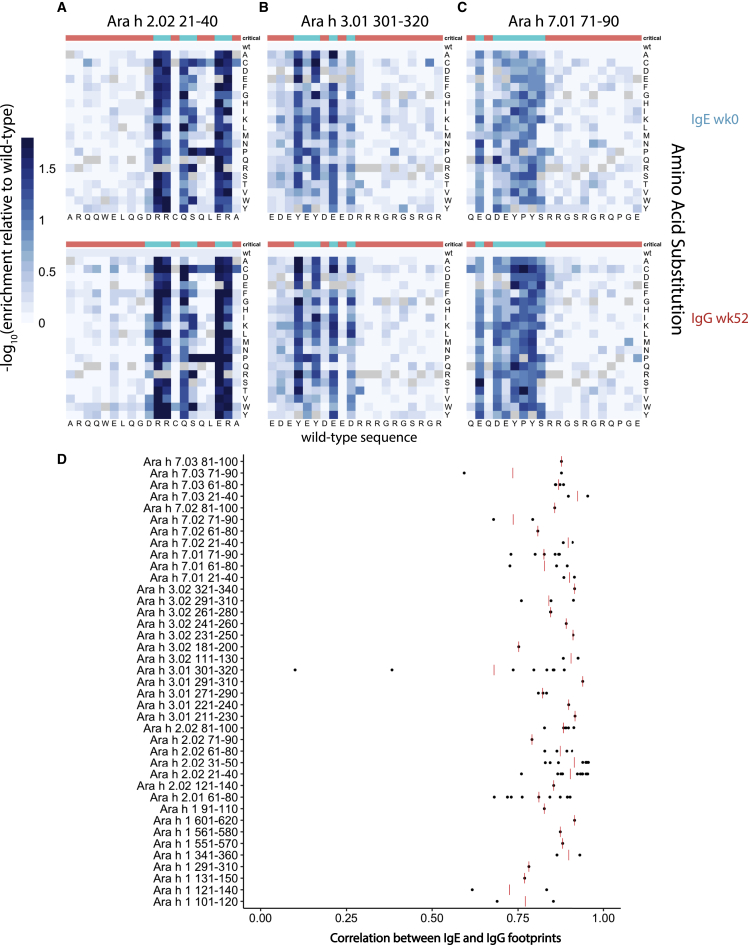
IgE and IgG antibody footprints for the same peanut epitopes are highly similar within individuals (A–C) Representative high-resolution antibody footprints as in Figures 2A–2C, comparing week 0 IgE (top row) and week 52 IgG (bottom row) from the same patient (B04) for peptides Ara h 2.02 aa21–40 (A), Ara h 3.01 aa301–320 (B), and Ara h 7.01 aa71–90 (C). (D) Pearson correlation coefficient between week 0 IgE and week 52 IgG footprints from the same patient for the peanut peptide indicated by the y axis. Each dot represents an IgE-IgG correlation coefficient for one patient. Red bar denotes the mean IgE-IgG correlation of all patients who are reactive to the given peptide.

As the IgE and IgG peptide-binding repertoires overlap substantially (Figure 4D), we wondered whether IgE and IgG bind epitopes with similar critical residue preferences. For this analysis, we compared the IgE and IgG footprints in epitopes shared by the week 0 IgE repertoire and the week 52 IgG repertoire, as these time points contain the larger epitope collection for IgE and IgG, respectively. Examination of the 3 representative peptides discussed earlier revealed that IgE and IgG footprints were in fact highly similar and, in some cases, almost indistinguishable (Figures 6A–6C and S4). To generalize this observation, we calculated the correlation between IgE and IgG footprints for every shared epitope for the same individual (Figure 6D). Strikingly, out of 39 peptides, all but 4 exhibited a mean IgE-IgG footprint correlation of >0.75. If each peptide recognized by an individual patient was considered a single observation, 91 out of 103 patient-epitope pairs (individual datapoints in Figure 6D) showed a high level of similarity between IgE and IgG footprints. Highly similar recognition of critical residues in the epitopes strongly suggest structural and sequence similarity, and perhaps clonal relatedness, between OIT-induced IgG antibodies and pre-existing peanut-specific IgE antibodies.

## Discussion

In this study, we report the synthesis of a comprehensive phage-display library of peanut-allergen-derived peptides to examine antibody repertoires in PA individuals. Due to the relative low cost of chip oligonucleotide synthesis and ease of generating highly diverse phage libraries, saturation mutants of each wild-type peanut peptide were included, enabling us to map antibody-epitope interactions at amino acid resolution. A readout via massively parallel sequencing enabled semiquantitative measurements of epitope-specific antibody abundance in serum. Using peanut AllerScan, we profiled the epitope specificities of the IgE and IgG repertoires of 15 PA children enrolled in a clinical trial of peanut OIT (PRROTECT).^17^ The confidence in the public epitopes we detected is strengthened by high reproducibility between technical replicates (usually with Pearson correlation of 0.95 or above), IgE signal detected across numerous allergic patients, but not NA donors, and quantitative enrichment of saturation mutants that allowed the construction of antibody footprints. To exclude the possibility that the IgE signal we detected is due to the polyreactive or promiscuous nature of IgE antibodies produced by allergic patients, we did not observe clear elevation of IgE epitopes detected from non-peanut species in our viral peptidome library^16^ compared to NA donors (data not shown). Importantly, this study confirmed many previously identified peanut peptides recognized by IgE antibodies from allergic patients,^18^^,^^22^^,^^23^ further supporting the robustness of AllerScan and the previous methodologies.

Surprisingly, Ara h 6, a major allergen homologous to Ara h 2, was only bound by IgE from 3/15 patients. Although Ara h 6 is generally recognized at a lower frequency than Ara h 2,^24^ the limited Ara h 6 response detected by AllerScan could also suggest a predominance of discontinuous or conformational epitopes. Evidence for the presence of both conformational and contiguous peanut epitopes has been presented previously using native and denatured proteins.^25^^,^^26^ However, the relative extent to which conformational epitopes are recognized by peanut-specific antibodies and contribute to pathogenesis or reversal of allergy has not been characterized. We look forward to the development of methods to comprehensively profile and map individual conformational epitopes to further explore this issue.

We and others^8^^,^^12^ found that OIT significantly increases peanut-specific IgG by several logs. Comparing post-OIT sera to pre-OIT, we found an increase in the number of IgG epitopes and a simultaneous increase in IgG-binding intensity for many epitopes that existed pre-OIT. Thus, this induction is driven by both diversification of anti-peanut IgG repertoire and increased concentration of individual IgG specificities. It has long been known that allergen-specific IgG antibodies can inhibit IgE-mediated immediate hypersensitivity. Three major mechanisms have been proposed to explain this effect: (1) steric interference with IgE binding to shared peanut epitopes;^6^^,^^12^ (2) formation of immune complexes that might accelerate allergen clearance; and (3) receptor-mediated inhibition of mast cell or basophil activation via binding to the inhibitory FcγRIIb receptor.^6^, ^7^, ^8^, ^9^ Using basophil activation as a readout, we have previously shown that receptor-mediated inhibition, exerted via the inhibitory IgG receptor FcgRIIb, is important in the allergen unresponsive state elicited by peanut OIT. Regardless of the major mechanism at play, the broader and stronger IgG responses to peanut epitopes we observed in patients post-OIT would be predicted to counter hypersensitivity. We also found that IgE epitope diversity remained largely constant after OIT, consistent with findings by Vickery et al.^27^ Further, the reduction in pre-existing epitope-specific IgE levels during the course of OIT is consistent with the overall decrease in peanut-specific IgE levels previously observed.^8^^,^^10^^,^^27^ In addition to repertoire level changes, we additionally noted a marked induction of Ara-h-7-specific IgG by OIT. While 11/15 allergic individuals had Ara-h-7-specific IgE pre-OIT, only 2 concurrently had Ara-h-7-specific IgG. Strikingly, all 12 individuals with Ara-h-7-specific IgE after OIT also had corresponding IgG. IgG binding of certain Ara h 7 epitopes increased up to 25-fold measured by *Z* score. It remains of interest to validate and further elucidate the association between Ara-h-7-specific IgG antibodies and successful desensitization to peanut.

Additionally, IgG epitopes that emerged during OIT were found to substantially overlap with pre-existing IgE specificities and share highly similar antibody footprints. This observation suggests that OIT-induced IgG could result from preferential stimulation of IgG B cells clonally related to peanut-specific IgE B cells. Affinity-matured IgG memory B cells have been suggested to replenish the pool of the IgE-producing plasma cells responsible for allergy in mice.^28^^,^^29^ Deep sequencing of immunoglobulin heavy chain genes (IGH) rearrangements in peripheral blood by Looney and colleagues supports such a clonal relationship between IgE and IgG B cell lineages in allergic human subjects.^30^ It is possible that affinity-matured IgG+ memory B cells preferentially differentiate into IgG-producing plasma cells during OIT. Another possibility is that the same immunodominant epitopes that induce peanut-specific IgE independently drive primary, convergent IgG responses. Future analyses could differentiate these two hypotheses by analyzing the clonal overlap between OIT-induced IgG B cells and pre-existing IgE B cells. In either case, repeated antigen exposure during OIT seems to preferentially stimulate IgG production over IgE to achieve the observed increase in peanut-specific IgG concentration and diversity. Future studies should aim to further elucidate pathway underlying this OIT-induced shift from IgE-dominant to IgG-dominant responses.

Using our peanut AllerScan platform, we identified extensively shared public epitopes in the major peanut allergens Ara h 1, 2, 3, and 7, with recognition frequency of up to 87% in our cohort of 15 allergic patients. Interestingly, the antibody footprints derived from saturation mutagenesis of these epitopes also showed a tremendous degree of similarity across individuals, suggesting structural and sequence similarity of public epitope-specific antibodies. In agreement with our finding, recent studies that performed B cell receptor (BCR) sequencing of peanut-specific B cells reported shared immunoglobulin heavy and light chain gene (IGH and IGL) segment usage and homologous CDR sequences among different PA individuals.^31^, ^32^, ^33^ The discovery of such public “clones” has been limited to Ara-h-2-specific B cells, mostly of non-IgE isotypes. Our identification of public IgE and IgG epitopes in the other major peanut allergens suggests that public antibodies are also generated toward Ara h 1, 2, 3, and 7. Importantly, Ara h 7 has only recently been recognized to be a major peanut allergen, with a discriminative ability similar to those of Ara h 2 and 6.^34^

Interestingly, Hoh et al.^32^ reported that convergent Ara h 2 antibody sequences are also present in NA participants but only in non-IgE isotypes, such as IgG. Although we cannot rule out the possibility that these public antibodies recognize a conformational epitope not captured by AllerScan, we did not detect IgG reactivity in NA sera to the peanut public epitopes recognized by allergic sera. In the rare occurrences of IgG reactivity to peanut epitopes in NA sera, the epitope specificities were not frequently shared with allergic sera. The only public IgG epitope (>30% reactivity) found in NA sera was not recognized by any patients in the PA group. These findings suggest that allergy is associated with a distinct set of IgG peanut epitopes than those from NA individuals. Another possibility is that many IgG epitopes we identified in NA sera are cross-reactive with antigens unrelated to peanut.

Although examples of sequence similarity between peanut-specific antibodies from different individuals and between IgE and IgG peanut-specific B cells from the same individual have previously been described,^31^, ^32^, ^33^^,^^35^ the application of BCR sequencing to study antibody repertoires is limited by the very low frequency of peanut-specific B cells in the peripheral blood. Furthermore, isolating peanut-specific IgE+ B cells highly relevant to allergy remains a formidable technical challenge as they make up a minute fraction of B cells in circulation. Only a small minority of IgE+ B cells isolated by fluorescence-activated cell sorting (FACS) are confirmed to be truly IgE+ upon BCR sequencing.^31^ Although the gastrointestinal (GI) tract harbors a larger number of IgE+ B cells,^32^ the number of cells that can be practically obtained from gastrointestinal biopsies is still insufficient to establish the entire IgE repertoire of one individual, let alone to make generalized conclusions about the population of PA individuals as a whole. We propose AllerScan as an alternative approach to investigate the IgG and IgE repertoires across populations and within individuals. Although AllerScan does not provide direct information about BCR sequences, the high-resolution antibody footprints we have generated provide valuable information about the similarity of the antigen-binding interface between antibodies that bind a given peptide. In most cases, the short length of peptides (20 aa) can ensure exclusive binding by one antibody (or a group of related antibodies). An additional advantage of AllerScan is high sensitivity of epitope detection requiring only <25 μL serum for IgE and <1 μL for IgG. Finally, AllerScan can be easily adapted to evaluate antibody responses to other allergens via the design of a new library.

The power of AllerScan to rapidly evaluate hundreds of sera samples in parallel at low cost could enable novel strategies for personalized immunotherapy. Our findings show that, despite shared recognition of public epitopes by allergic patients, the exact constellation of public epitopes recognized by any individual varied. For instance, out of 20 peptides recognized by IgE of at least 5/15 allergic patients pre-OIT, each patient lacked IgE responses to 4–15 (average = 9) peptides. We envision that the personalized identification of sets of “missing” public epitopes could inform a novel and much safer type of allergen immunotherapy. In this approach, the set of peptides containing missing public epitopes would be formulated into a peptide vaccine designed to elicit protective IgG responses. In the absence of pre-existing IgE responses to these peptides, the risk for allergic reactions would be greatly minimized compared to whole-peanut OIT.

In summary, our findings reveal that the phage immunoprecipitation platform, AllerScan, provides an innovative, precise, and efficient approach for investigating antibody responses in allergic subjects. Peptide phage display has also been applied to wheat allergies in a recent study.^36^ Here, we showed that, together with saturation mutagenesis, AllerScan creates a high-resolution image of both IgG and IgE repertoires and can be used to track their evolution during immunotherapy for allergy. We propose that the detailed definition of both epitopes recognized by the antibodies of food-allergic subjects and epitopes that are not may have practical applications in future personalized allergen immunotherapy strategies.

### Limitations of the study

Although we were able to identify many peanut epitopes using AllerScan, we acknowledge the limitation that discontinuous epitopes might not be detected using peptides. The existence of conformational epitopes in peanut allergy is suggested by a number of studies, but the relative contributions of conformational versus linear epitopes to IgE-mediated food reactions remain unclear. We look forward to the development of new technologies capable of characterizing and quantifying conformational epitope binding to aid in the investigation of this question. Although our study identified a strong induction of Ara-h-7-specific IgG by OIT, the clinical relevance of this IgG response will require further investigation. We have suggested that the high degree of similarity between the IgE and IgG footprints within individuals indicates that OIT-induced IgG and IgE B cells originate from the same lineages, but the alternative possibility that the overlapping IgE and IgG responses arise from convergent immunodominant responses is not excluded by our analysis and will be the subject of future studies.

## STAR★Methods

### Key resources table

**Table:** 

REAGENT or RESOURCE	SOURCE	IDENTIFIER
**Antibodies**		

Anti-human IgE antibody, biotinylated	Southern Biotech	Cat#9160-08; RRID: AB_2796671
Anti-human IgG antibody, biotinylated	Southern Biotech	Cat#9042-08; RRID: AB_2796608

**Biological samples**		

OIT allergic sera	MacGinnitie et al.^17^	Clinical trial NCT01781637, https://www.clinicaltrials.gov

**Critical commercial assays**		

Human IgE ELISA kit	Bethyl	Cat#E80-108

**Deposited data**		

Z-score data	This paper	Mendeley Data: https://doi.org/10.17632/zp97585dkp.1

**Oligonucleotides**		

T7-PFA aatgatacggcggGAATTCCGCTGCGT	Xu et al.^16^	N/A
T7-PRA caagcagaagACTCGAGCTCTTCCCTG	Xu et al.^16^	N/A

**Recombinant DNA**		

Peanut saturation mutagenesis AllerScan library	This paper	Annotations are available in Table S1 and are deposited at Mendeley Data: https://doi.org/10.17632/zp97585dkp.1
T7FNS2 vector	Larman et al.^15^	N/A

**Software and algorithms**		

Bowtie	Langmead et al.^37^	http://bowtie-bio.sourceforge.net/index.shtml
Cutadapt	Martin^38^	https://cutadapt.readthedocs.io/en/stable/
R, version 3.6.1		N/A
GraphPad Prism version 9.1.2		N/A
Samtools version 1.3.1	Li et al.^39^	http://www.htslib.org
tidyverse	Wickham et al.^40^	CRAN: tidyverse; RRID: SCR_019186

**Other**		

Pierce Streptavidin Magnetic Beads	Thermo Fisher Scientific	Cat#88817
T7 Select Packaging Kit	EMD Milipore	Cat#70014-3

### Resource availability

#### Lead contact

Further information and requests for resources and reagents should be directed to the Lead Contact, Stephen J. Elledge (selledge@genetics.med.harvard.edu).

#### Materials availability

All unique/stable reagents generated in this study are available from the Lead Contact with a completed Materials Transfer Agreement. Please direct resource and reagent requests to the Lead Contact specified above, Stephen J. Elledge.

### Experimental model and subject details

#### Human subjects

Allergic sera used in this study were originally collected in PRROTECT, a multicenter randomized placebo-controlled study of oral peanut desensitization under cover of anti-IgE (omalizumab) in highly allergic subjects (NCT01781637, https://www.clinicaltrials.gov). The study was approved by the Institutional Review Boards of all participating institutions^17^. Patients reacting to < 50 mg peanut protein were randomized 3.5:1 to receive either omalizumab or placebo for 12 weeks followed by rapid escalation to 250 mg of peanut protein on the first day of desensitization. Patients underwent weekly up-dosing over the next 8 weeks to a maximum dose of 2000 mg. Patients continued omalizumab or placebo for a total of 19 weeks, at which time drug was discontinued and OIT was continued. Placebo subjects failing to reach the 250 mg dose at 19 weeks were given the option to switch to open-label omalizumab and all but one opted to cross over to treatment with omalizumab prior to completing OIT. At 52 weeks (8 months off omalizumab), ten patients still could ingest 4000 mg peanut, up from less than 50 mg pre-OIT. The lowest tolerated dose was 1500 mg. Available patient age, gender, baseline total IgE and peanut-specific IgE information is provided in Table S6.

### Method details

#### Design and cloning of the peanut AllerScan library

397 20-mer wild-type peanut peptides were designed by tiling every 10 amino acids across 12 peanut allergens (Table S1). Saturation mutants were designed by replacing each amino acid at a given position in the wild-type peptide with every other possible amino. The peptide sequences were reverse-translated into DNA sequences and codon-optimized for expression in *Escherichia coli*. Synonymous mutations were made as needed to exclude restriction enzyme sites used for cloning and to ensure that the 50 nt at the 5′ end of each sequence were unique so that all sequences could be mapped unambiguously after high-throughput sequencing. We added adaptor sequences “GGAATTCCGCTGCGT” to the 5′ end and “CAGGGAAGAGCTCGA” to the 3′ end to form the final oligonucleotide sequences. The final oligonucleotide library was synthesized on releasable DNA microarrays by Agilent. The DNA oligo library was PCR amplified with T7-PFA (aatgatacggcggGAATTCCGCTGCGT) and T7-PRA (caagcagaagACTCGAGCTCTTCCCTG) primers, digested with EcoRI and XhoI, and ligated into the EcoRI/SalI site of the T7FNS2 vector^15^. T7 Select Packaging Kit (EMD Millipore) was used for T7 bacteriophage packaging. The phage library was amplified according to the manufacturer’s protocol.

#### Phage immunoprecipitation and sequencing

Phage immunoprecipitation and sequencing was performed as described previously^16^ with some modifications. The peanut phage display library was diluted such that each member of the library was present at approximately 2E5-fold representation in 1 mL reaction volume, and appropriate amounts of serum were added. For IgE IP reactions, serum containing 10 ng of total IgE, determined by ELISA, was added; for IgG IP, serum containing 2 ug of total IgG was added. Following overnight incubation of phage and serum at 4°C, biotinylated mouse anti-human IgE (Southern Biotech) or anti-human IgG (Southern Biotech) antibodies were added, and the mixture was incubated at room temperature for 4 h. Pierce Streptavidin Magnetic Beads (Thermo) was then added followed by incubation at 4°C overnight. For IgE IP, 10ug of anti-human IgE and 40 uL of Streptavidin beads were used; for IgG IP, 6 ug of anti-human IgG and 25 uL of Streptavidin beads were used. Subsequent washing and amplicon library preparation steps were performed as described in Xu et al.^16^. Mock-immunoprecipitation (mock IP) reactions (no serum added) were included and sequenced in all experiments. Each sample was sequenced to an average depth of 2 million reads. The sequencing reads were aligned to the library sequences using Bowtie and the read counts for each library member were tallied using samtools. We performed at least 2 technical replicates of each sample and routinely achieved above 0.95 Pearson correlation between replicates. Raw Z-score data can be found in Table S5 and have been uploaded to Mendeley Data associated with this manuscript (https://doi.org/10.17632/zp97585dkp.1).

### Quantification and statistical analysis

Statistical analysis was performed using either GraphPad Prism version 9.1.2 or R version 3.6.1. Corresponding statistical details are indicated in figure legends. Detailed bioinformatic analyses of AllerScan data were performed as follows.

#### Statistical analysis of AllerScan data

For every wild-type peanut epitope, we calculated Z-scores representing the relative enrichment value compared to mock IP reactions as previously described^41^. Briefly, peptides with identical or similar abundance in the input library (obtained by sequencing mock-IP reactions) were grouped into bins. The Z-scores for each peptide was calculated based on the null distribution of the bin where it resides, with top and bottom 5% removed, using the following formula:

$$Z=\frac{\left[peptide\;read\;counts\right]-\;mean}{stdev}$$

where [peptide read counts] is the number of reads for the peptide of interest, and the “mean” and “stdev” are the mean and standard deviation of read counts for the middle 90% of the bin to which the peptide of interest was assigned based on the mock IP input distribution. More details on this method have been described in Mina et al.^41^, where we have also shown that the Z-scores calculated using this method achieves relatively uniform ability to detect enrichment of peptides regardless of input phage abundance. The Z-score was also shown to be a quantitative measure of epitope-specific antibody levels in plasma.

#### Quantification of repertoire breadth

To quantify the breadth of antibody repertoires (Figure 4B), we first excluded genetic and splice variants of each Ara h protein to avoid counting the same epitope multiple times. We calculated the mean Z-scores of each remaining epitope across at least 2 replicates. We then set mean Z-score > 3.5 as the threshold for calling an epitope reactive and enumerated the total number of reactive epitopes in each sample.

#### Substitution effects and critical residue analysis

To assess the effects of amino acid substitutions on antibody binding to a given peptide, we first estimated the wild-type peptide enrichment by calculating the mean enrichment of the wild-type peptide and the top 50% of the alanine mutants of that peptide. We found that this value was a more robust estimate of the true enrichment of the wild-type peptide than the enrichment value of the single wild-type peptide alone. Then, the enrichment of each mutant peptide was divided by the estimated enrichment of wild-type peptide as calculated above. This became the relative-to-wild-type enrichment. This process was repeated for all saturating mutants of a given wild-type peptide. To generate a high-resolution antibody footprint on an epitope (Figures 2A–2C and 4A–4C), we plotted the -log10 (1 / relative-to-wild-type enrichments) values for all mutants, representing their respective substitution effects. A darker color represented greater disruption to antibody binding. A residue was considered “critical” if the median enrichment of the 19 mutants at this position was less than 40% of the wild-type estimate.

To visualize the substitution effect at each amino acid position (Figures 2, S2, and S3), we plotted the mean depletion of the 19 mutants relative to the wild-type estimate at that position (1 – mutant / wild-type). We limited this analysis to wild-type peptides with Z-scores > 5. If overlapping peptides both had Z-scores > 5, the average substitution score was taken for the overlapping residues. Replicates were analyzed individually and results from one replicate were plotted.

### Additional resources

Information on PRROTECT clinical trial (registry number NCT01781637): https://clinicaltrials.gov/ct2/show/NCT01781637

## Data Availability

Processed sequencing data (as Z-scores) have been uploaded to Mendeley Data and are publicly available as of the date of publication. DOIs are listed in the Key resources table. We do not report custom code. Any materials needed to analyze data in this work are available upon request from the Lead Contact. Any additional information required to reanalyze the data reported in this work is available from the Lead Contact upon request.

## Supplemental information

Document S1. Figures S1–S6 and Table S6

Table S1. Peanut AllerScan library annotations, related to Figure 1 and STAR Methods Sequences of wild-type and mutant 20-mer peanut peptides.

Table S2. IgE epitopes and recognition frequency in peanut-allergic patients, related to Figure 1

Table S3. IgG epitopes and recognition frequency in peanut-allergic patients, related to Figure 1

Table S4. Sample information and index for Table S5, related to STAR Methods

Table S5. *Z* scores for all samples and technical replicates used in this study, related to STAR Methods

Document S2. Article plus supplemental information
