# Supplementary material for: High-resolution epitope mapping by AllerScan reveals relationships between IgE and IgG repertoires during peanut oral immunotherapy
Source: Cell Rep Med. 2021 Oct 19;2(10):100410. doi: 10.1016/j.xcrm.2021.100410 (PMC8563412; doi:10.1016/j.xcrm.2021.100410)
Supplement: Document S1. Figures S1–S6 and Table S6 [file mmc1.pdf]

Cell Reports Medicine, Volume *2*

Supplemental information

High-resolution epitope mapping by AllerScan reveals relationships between IgE and IgG repertoires during peanut oral immunotherapy

Genghao Chen, Ellen L. Shrock, Mamie Z. Li, Jonathan M. Spergel, Kari C. Nadeau, Jacqueline A. Pongracic, Dale T. Umetsu, Rima Rachid, Andrew J. MacGinnitie, Wanda Phipatanakul, Lynda Schneider, Hans C. Oettgen, and Stephen J. Elledge

Supplementary Figures

Supp. Figure 1

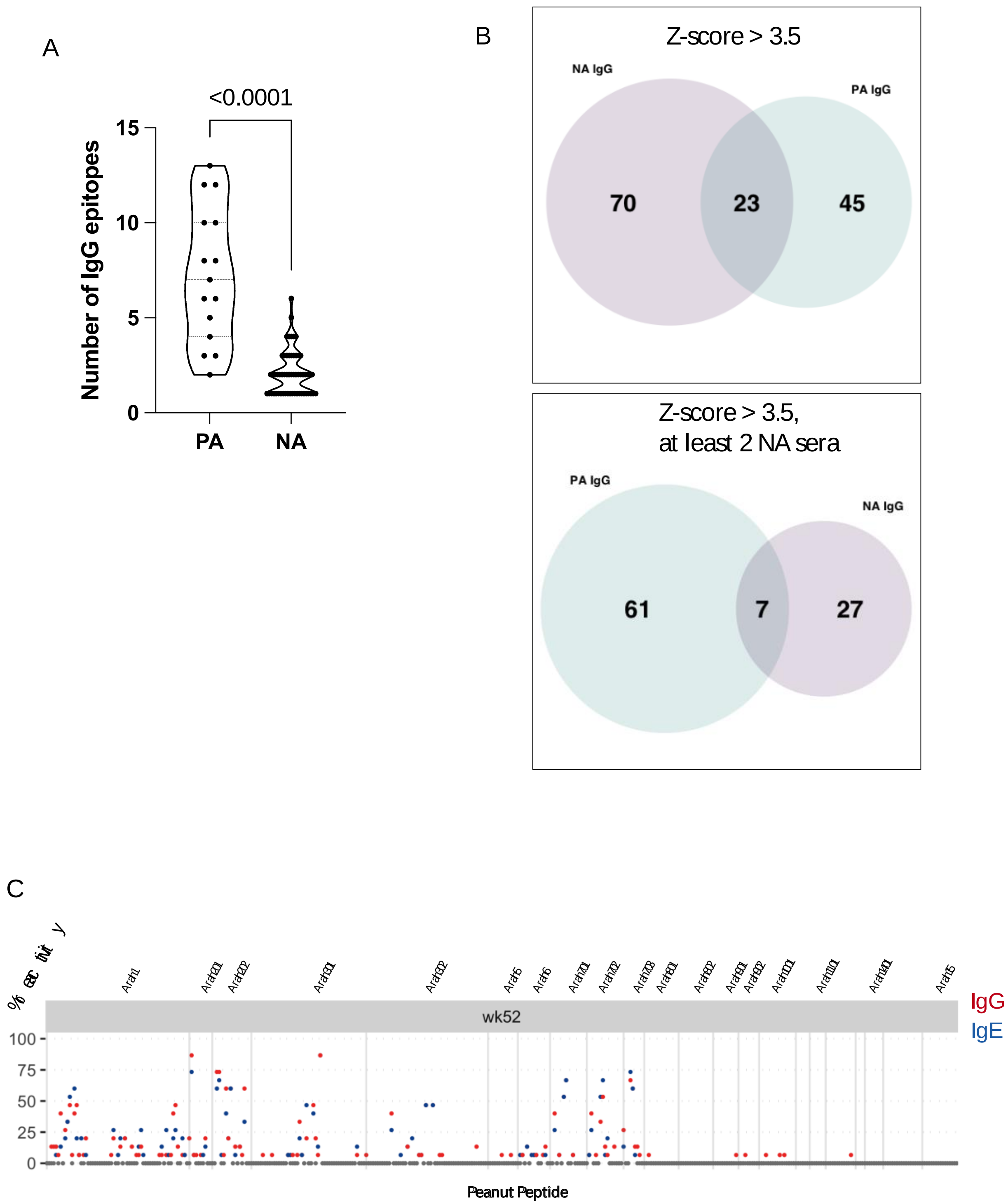

Figure S1. Quantification of IgG epitopes recognized by peanut-allergic vs non-allergic individuals. Related to Figure 1. (A) Number of IgG epitopes recognized by peanut-allergic (PA) OIT patients (week 0 sera) and non-allergic (NA) sera. P-value = 5.1e-6, Student's t test. (B) Overlap between PA and NA epitopes with Z-scores > 3.5 (top) or Z-scores > 3.5 in at least 2 NA sera (bottom). (C) Seroprevalence of antibodies to peanut epitopes in week 52 (after OIT). Percentage of allergic patients exhibiting IgE (blue) and IgG (red) reactivity to peptides in the peanut AllerScan library.

Supp. Figure 2

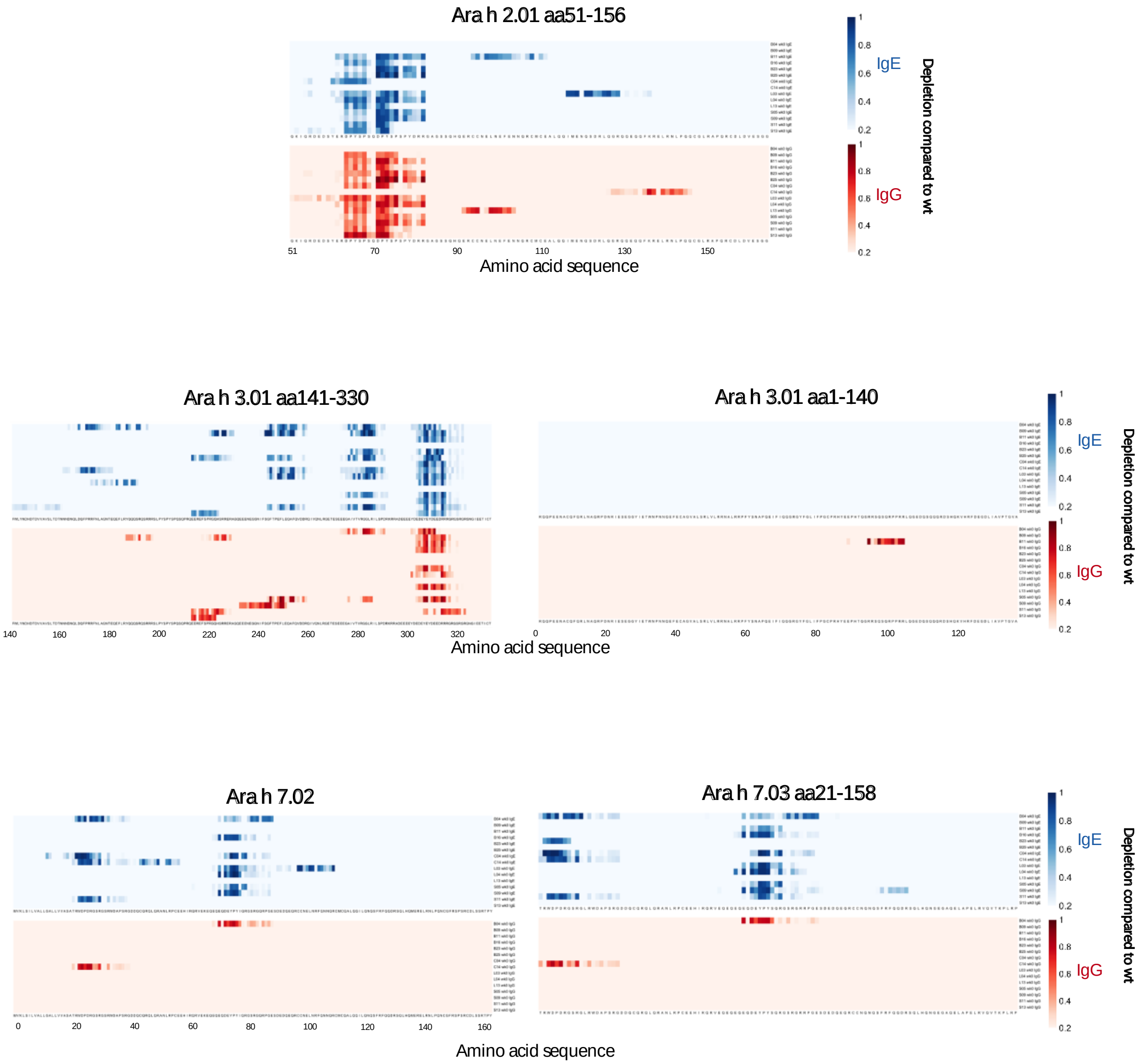

Figure S2. Critical residues of IgE and IgG epitopes (OIT week 0 sera). Related to Figure 2. Heatmaps as in Figure 2 show detailed mapping of IgE and IgG epitopes in OIT patients at week 0.

Supp. Figure 3

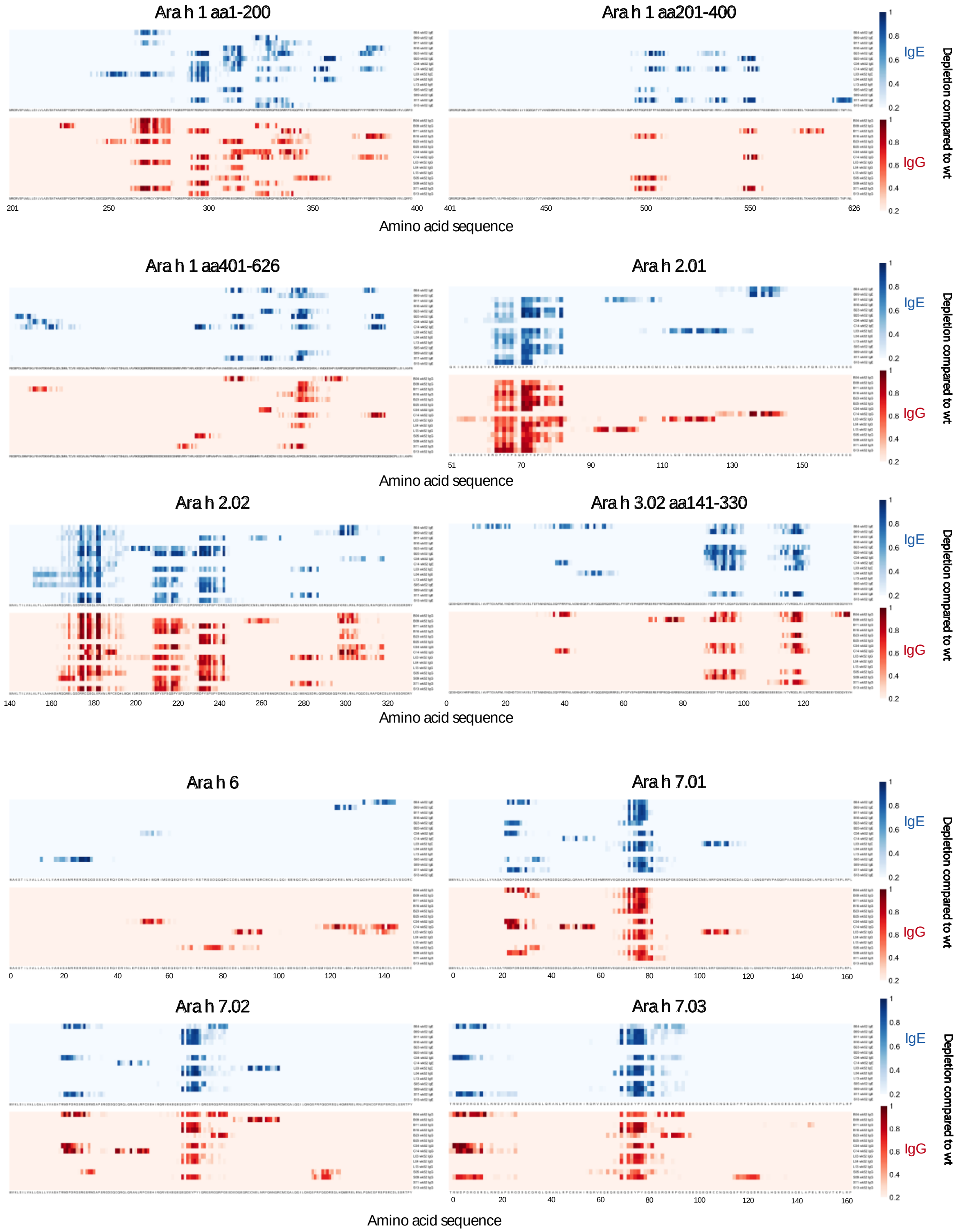

Figure S3. Critical residues of IgE and IgG epitopes (OIT week 52 sera). Related to Figure 2. Heatmaps as in Figure 2 show detailed mapping of IgE and IgG epitopes in OIT patients at week 52.

Supp. Figure 4

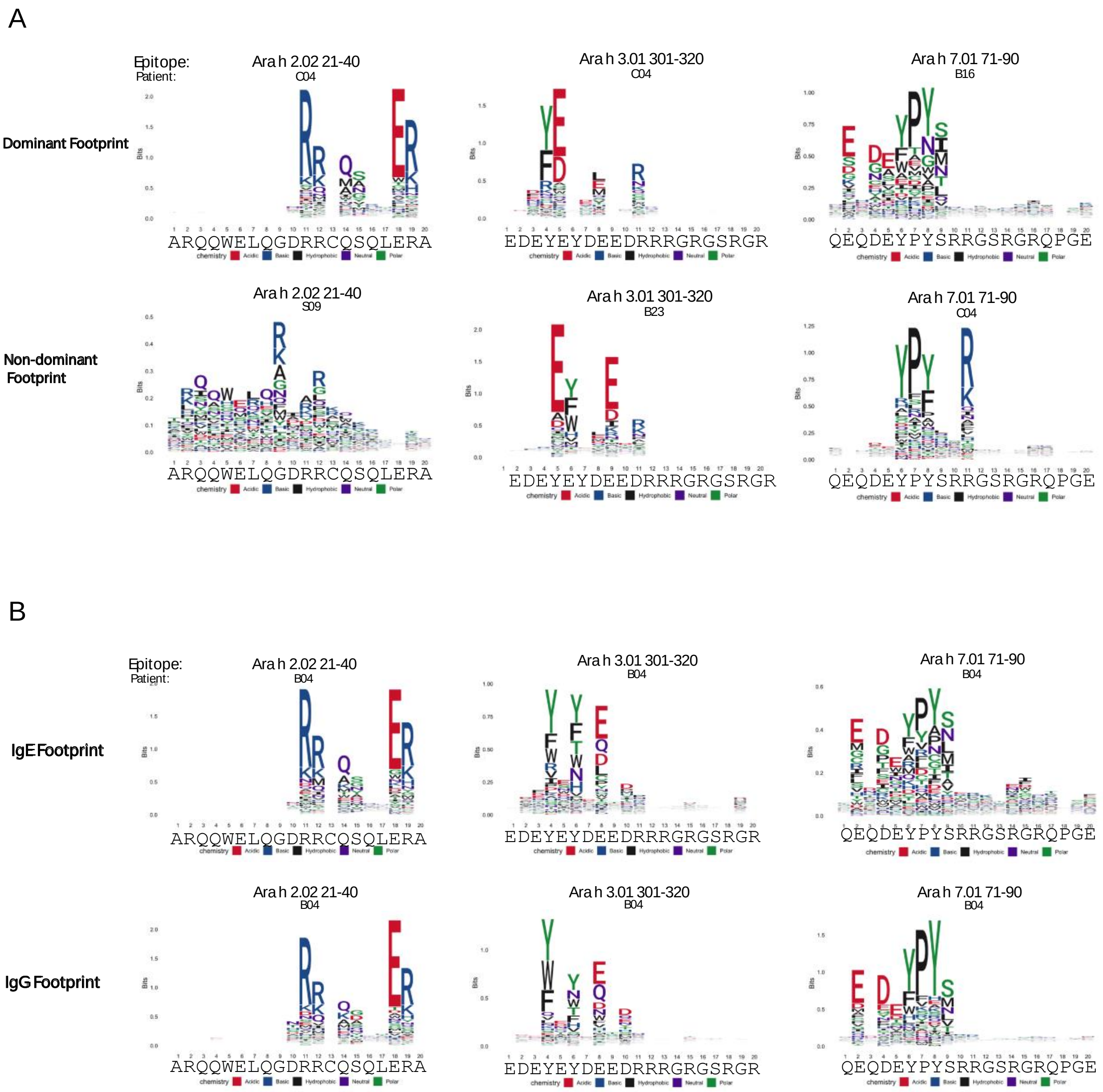

Figure S4. Sequence logo plots illustrate permissibility of amino acid substitutions in public epitopes and similarity between one individual's IgE and IgG footprints. Related to Figure 3. Each logo plot shows the permissible amino acids at the indicated positions of a peanut public epitope for one allergic patient. (A) Dominant (top) footprints and non-dominant (bottom) footprints of IgE epitopes obtained from pre-OIT sera. (B) IgE (top) and IgG (bottom) antibody footprints from one allergic patient, B04, for three public epitopes are depicted. IgE footprints were generated from week 0 sera and IgG footprints were generated from week 52 sera. To generate sequence logo plots, relative-to-wild-type enrichment matrices were used as inputs to the ggseqlogo R package [22].

Supp. Figure 5

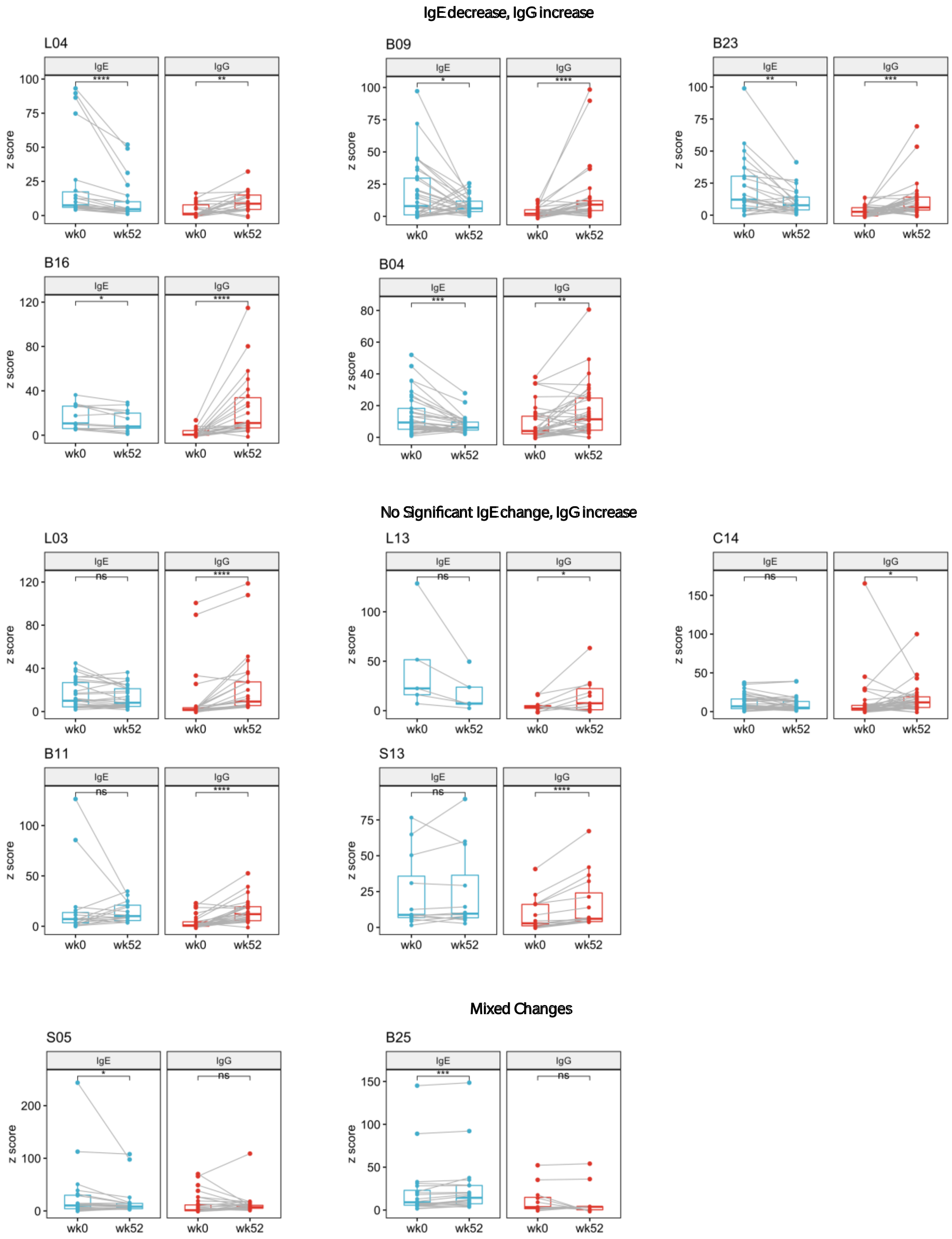

Figure S5. OIT-induced changes in peptide-specific IgE and IgG reactivity. Related to Figure 4 and 5. Change in peptide binding Z-score, from week 0 to week 52 in allergic patients. Only peptides with week 0 or week 52 reactivity (Z-score >3.5) were examined. Each dot represents one peptide. Top, patients exhibiting overall reduction in IgE and increase in IgG reactivity to peanut peptides; middle, patients with no change in IgE and increase in IgG; bottom, patients with mixed changes in IgE and IgG binding. Statistical significance was determined by Wilcoxon signed rank test.

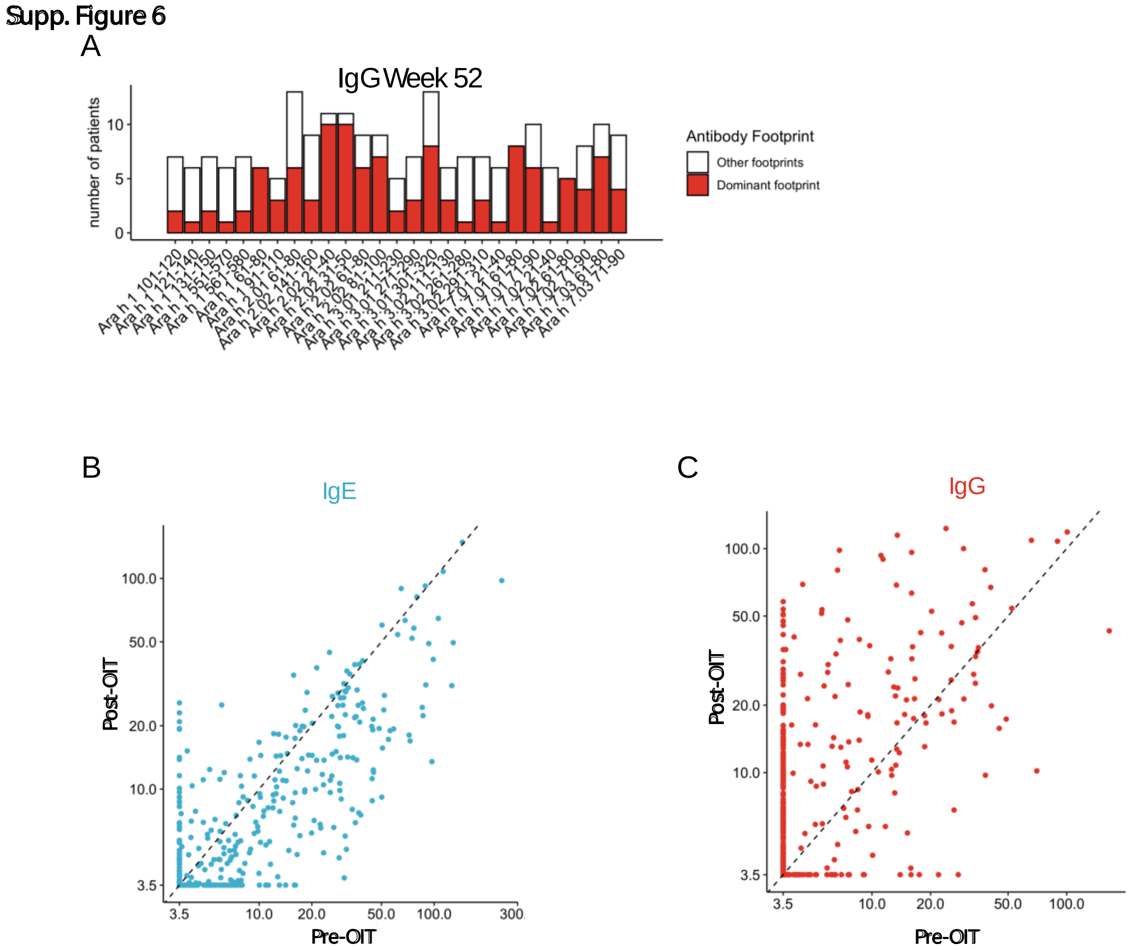

Figure S6. Similarity of IgG footprints among post-OIT sera and scatterplots comparing pre-OIT and post-OIT peptide binding by IgE and IgG. Related to Figure 3, 4 and 5. (A) Number of patients who share the dominant IgG antibody footprint for each of the public epitopes indicated by the x-axis after OIT (week 52). Red, dominant footprint; white, other footprints. (B, C) Scatterplots comparing pre-OIT and post-OIT Z-scores, a surrogate measure of peptide-specific antibody titer, of (B) IgE and (C) IgG epitopes. Each dot represents one peptide. Peptides with Z-score < 3.5 were collapsed to Z = 3.5. Diagonal line represents unchanged Z-scores.

Supplementary Table Legends

Table S6. Patient clinical information. Related to STAR Methods.

Patient ID	Sample group	Age	Gender	Baseline Total IgE (kUa/L)	Baseline peanut-specific IgE (kUa/L)
L13	OIT	7	F	224	27.0
B04	OIT	15	M	478	147.0
B09	OIT	9	M	493	162.0
B11	OIT	11	M	282	90.6
B16	OIT	14	F	1055	54.0
C04	OIT	7	M	125	26.1
L04	OIT	10	M	177	41.8
S05	OIT	10	M	858	296.0
S09	OIT	9	M	798	251.0
S13	OIT	7	M	661	483.0
B25	OIT	19	F	997	263.0
L03	OIT	11	F	953	360.0
S11	OIT	8	F	207	61.1
B23	OIT	9	F	918	535.0
C14	OIT	15	F	491	273.0
**OIT MEAN**	OIT	10.7	N/A	581.1	204.7
**OIT SD**	OIT	3.5	N/A	331.0	164.9
EASY-9018	non-allergic	9	F	224	<0.35
EASY-9027	non-allergic	9	M	478	<0.35
EASY-9038	non-allergic	9	F	493	<0.35
EASY-9058	non-allergic	8	M	282	<0.35
**EASY MEAN**	non-allergic	8.8	N/A	369.3	<0.35
**EASY SD**	non-allergic	0.5	N/A	136.4	N/D

We do not have information on the remaining non-allergic healthy donors.
